# Case report: Triple-negative breast cancer with low tumour-infiltrating lymphocytes infiltration and good prognosis: a case of Tall Cell Carcinoma with Reversed Polarity and review of the literature

**DOI:** 10.3389/fonc.2024.1455893

**Published:** 2024-11-18

**Authors:** Shuoying Jiao, Junpeng Chen, Jiumei Shen, Ajing Peng, Ruifu Chen, Baoyong Lai, Chunhua Luo, Yingyi Fan, Xiaohua Pei

**Affiliations:** ^1^ Department of Breast, The Third Affiliated Hospital of Beijing University of Chinese Medicine, Beijing, China; ^2^ Department of Breast Surgery, Xiamen Hospital of Traditional Chinese Medicine, Xiamen, China; ^3^ Department of Pathology, Xiamen Hospital of Traditional Chinese Medicine, Xiamen, China

**Keywords:** triple-negative breast cancer, Tall Cell Carcinoma with Reversed Polarity, tumour-infiltrating lymphocytes, cancer-associated fibroblasts, case report

## Abstract

**Background:**

Triple-negative breast cancer (TNBC) is a great challenge for clinical management due to the high rate of metastatic recurrence and lack of recognised therapeutic targets, while tumour-infiltrating lymphocytes (TILs) infiltration in TNBC has been discovery provides an opportunity for immunotherapy, and studies have suggested that the level of TILs infiltration is positively correlated with TNBC survival. We found a rare case of TNBC with low TILs infiltration but good prognosis, named Tall Cell Carcinoma with Reversed Polarity (TCCRP), and we investigated it from the perspective of TILs infiltration in order to improve the clinical data of TCCRP.

**Case presentation:**

a 54-year-old woman with a left breast mass, breast ultrasound showed BI-RADS IVb, puncture diagnosis of invasive ductal carcinoma, postoperative pathology showed papillary structure, cubic and high columnar arrangement of cells, nuclei far away from the base, interstitial fibroplasia, diagnosed as TCCRP. Immunohistochemistry revealed that the tumours were triple-negative breast cancer (negative for ER, PR, and HER-2), with a low Ki-67 proliferation index. TILs were < 10%, with a small infiltration of CD4^+^ and CD8^+^ T lymphocytes, positive expression of SMA and FAP, and IDH2, PIK3CA gene mutation. The patient underwent postoperative chemotherapy, 11 months follow-up, no recurrence and metastasis.

**Conclusion:**

TCCRP is a rare TNBC with inert biological behaviours and good prognosis. We found low infiltration of TILs in the pathological tissue of this case, which may be a characteristic of TCCRP, and the presence of Cancer-Associated Fibroblasts (CAF) in the interstitium of the tumour in this case may have suppressed the anti-tumour immunity to some extent, and further studies on the immune characteristics of the tumour microenvironment (TME) in TCCRP are needed.

## 1 Introduction

Breast cancer is currently the second most prevalent cancer in the world, accounting for 11.6% of all cancers globally, and the fourth leading cause of cancer deaths worldwide (1). Triple-negative breast cancer (TNBC), a subgroup of breast cancer defined by the lack of expression of oestrogen receptor, progesterone receptor, and human epidermal growth factor receptor 2, accounts for 15 to 20% (2). Clinical management of TNBC is a great challenge due to the high incidence of visceral metastases and the lack of recognised therapeutic targets (3). In recent years, immunotherapy has emerged as a promising treatment modality for TNBC (4). The tumour microenvironment (TME) is immunologically complex and involves the interaction of multiple immune cells. Among them, tumour-infiltrating lymphocytes (TILs) have become a hotspot of interest in clinical studies on the immune aspects of TNBC (5), and although breast cancer is not considered an immunothermal tumour, a large infiltration of TILs can be found in TNBC (6). TILs include T cells, B cells and natural killer cells (NK), and in T cells, CD8^+^ cytotoxic T cells (CTL) are essential for tumour destruction, CD4^+^ T helper 1 (Th1) cells secrete cytokine mediators that help CTL improve toxicity. The level of TILs has an important predictive value in TNBC, and a study published in the Lancet noted that an increase in TILs was associated with prolonged overall survival in TNBC patients (7). Interestingly, we identified a case of a rare TNBC infiltrated by low TILs but with a good prognosis, called Tall Cell Carcinoma with Reversed Polarity (TCCRP). We first examined the expression of CD4^+^ T cells and CD8^+^ T cells and tried to analyse this case from the perspective of TILs infiltration to further improve the clinical data of this rare disease and provide ideas for treatment.

## 2 Case presentation

### 2.1 Case characteristic

**Figure 1:**
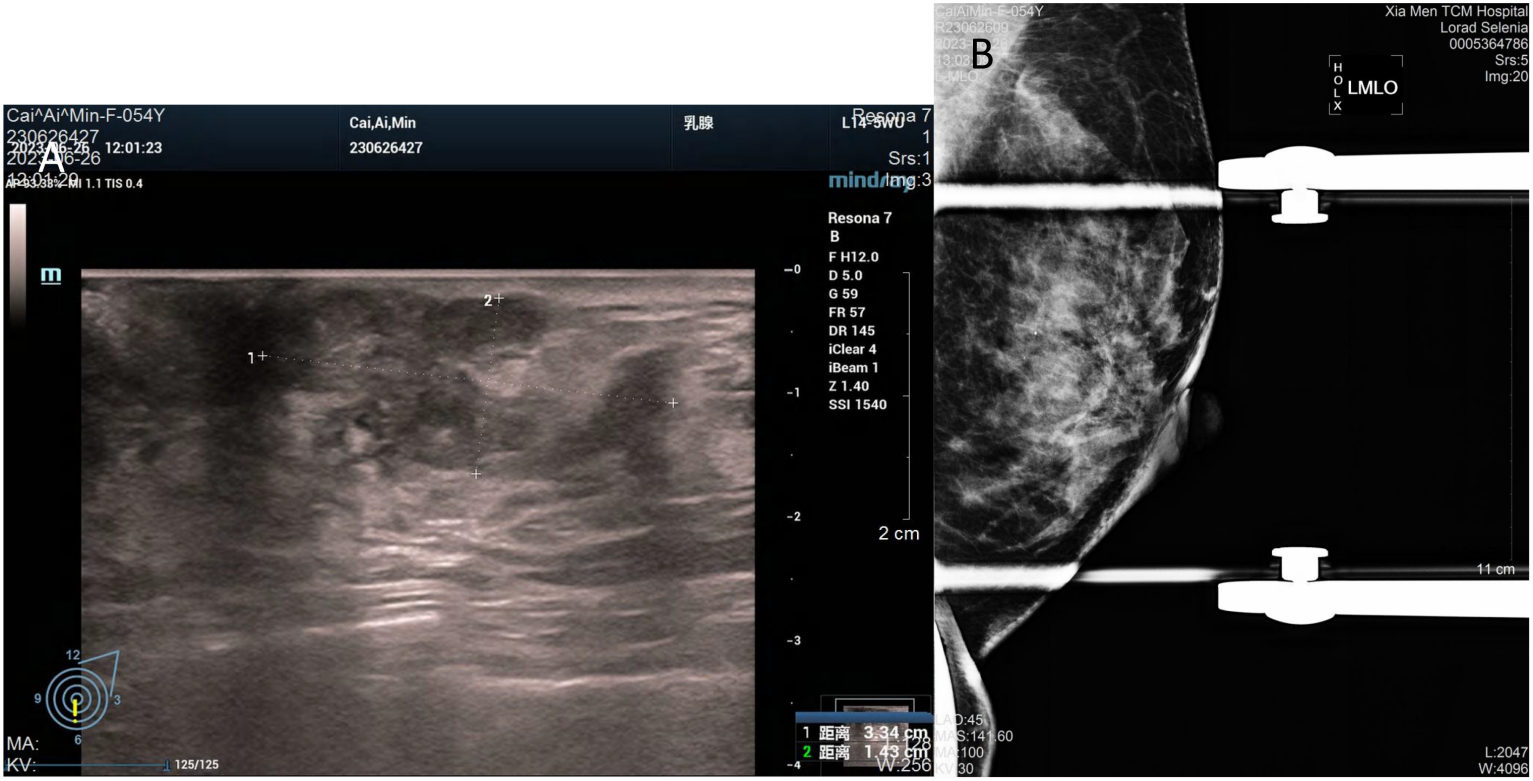
Imaging of the tumour. **(A)** Ultrasound shows an irregular, solid hypoechoic mass measuring 33mm×14mm with angular margins next to the nipple at 6 o’clock in the left breast. **(B)** Mammogram shows benign changes in both breasts, no mass was seen.

**Figure 2:**
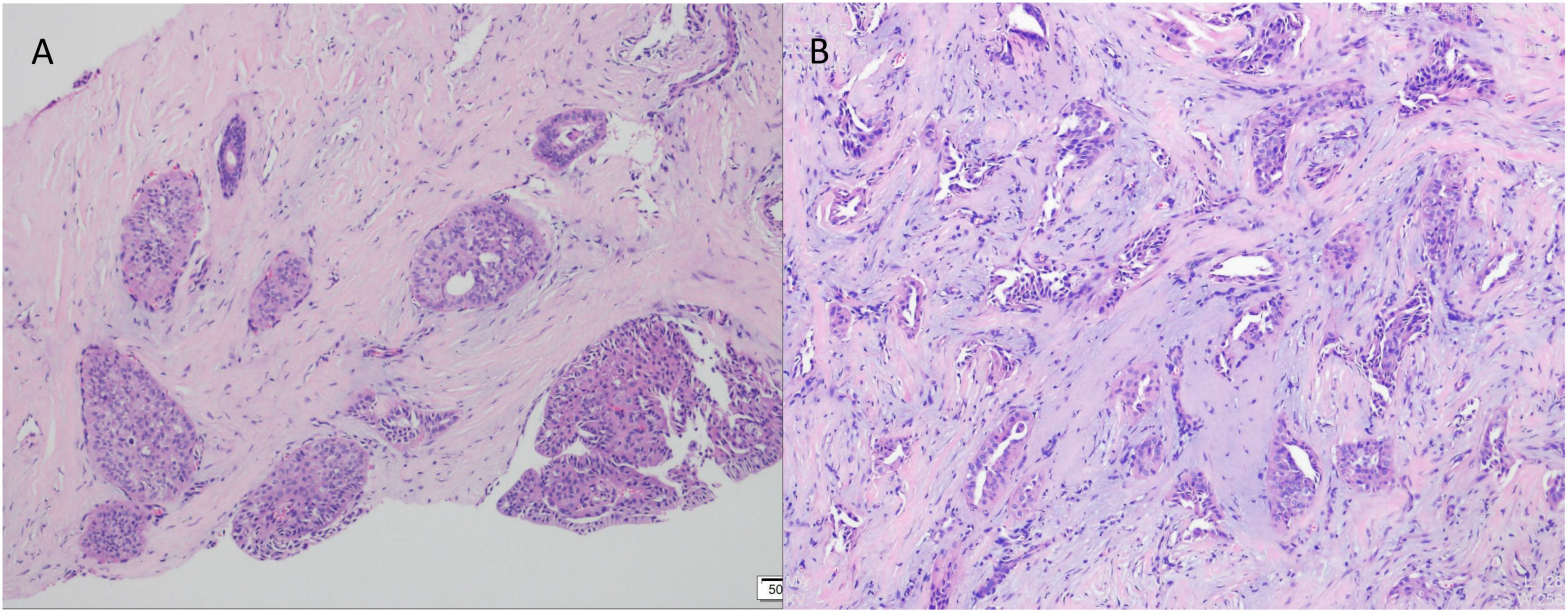
Pathological examination by puncture using haematoxylin and eosin (HE) staining. **(A)** Cancer cells were arranged in solid, papillary structures (×100). **(B)** Cancer cells arranged in an irregular adenoidal structure with infiltrative growth, tumour cells were columnar to cuboidal with mild nuclear morphology and acidophilic cytoplasm. Interstitial fibrous tissue hyperplasia and mucoid changes (×200).

In July 2023, a 54-year-old woman presented with a 10-year history of a left breast mass. The patient had discovered a left breast mass 10 years earlier without systematic treatment, and recently went to the hospital for examination after realising that the mass had increased in size compared to the previous one. Physical examination revealed well-developed and symmetrical breasts Physical examination revealed well-developed and symmetrical breasts without dimpling or skin changes resembling an orange peel. No bleeding or discharge was observed upon bilateral nipple compression. A mass was palpable just below the left areola, measuring 3.0cm×2.0cm, hard texture, unclear borders, poor mobility, no enlarged lymph nodes were detected in the axilla and clavicle bilaterally. There was no family history of breast cancer in the patient. Ultrasound showed a solid hypoechoic mass (BI-RADS: IVb) (**Figure 1A**). Mammogram showed benign changes in the glands of both breasts (BI-RADS:2) (**Figure 1B**), and no mass was seen. Puncture biopsy was performed, and the cancer cells were arranged in solid, papillary or irregular glandular structures (**Figures 2A, B**), and invasive ductal carcinoma was diagnosed, left mastectomy with sentinel lymph node biopsy was done, and the intraoperative pathology reported no metastasis of cancer macrosomes in lymph nodes (0/7).

**Figure 3:**
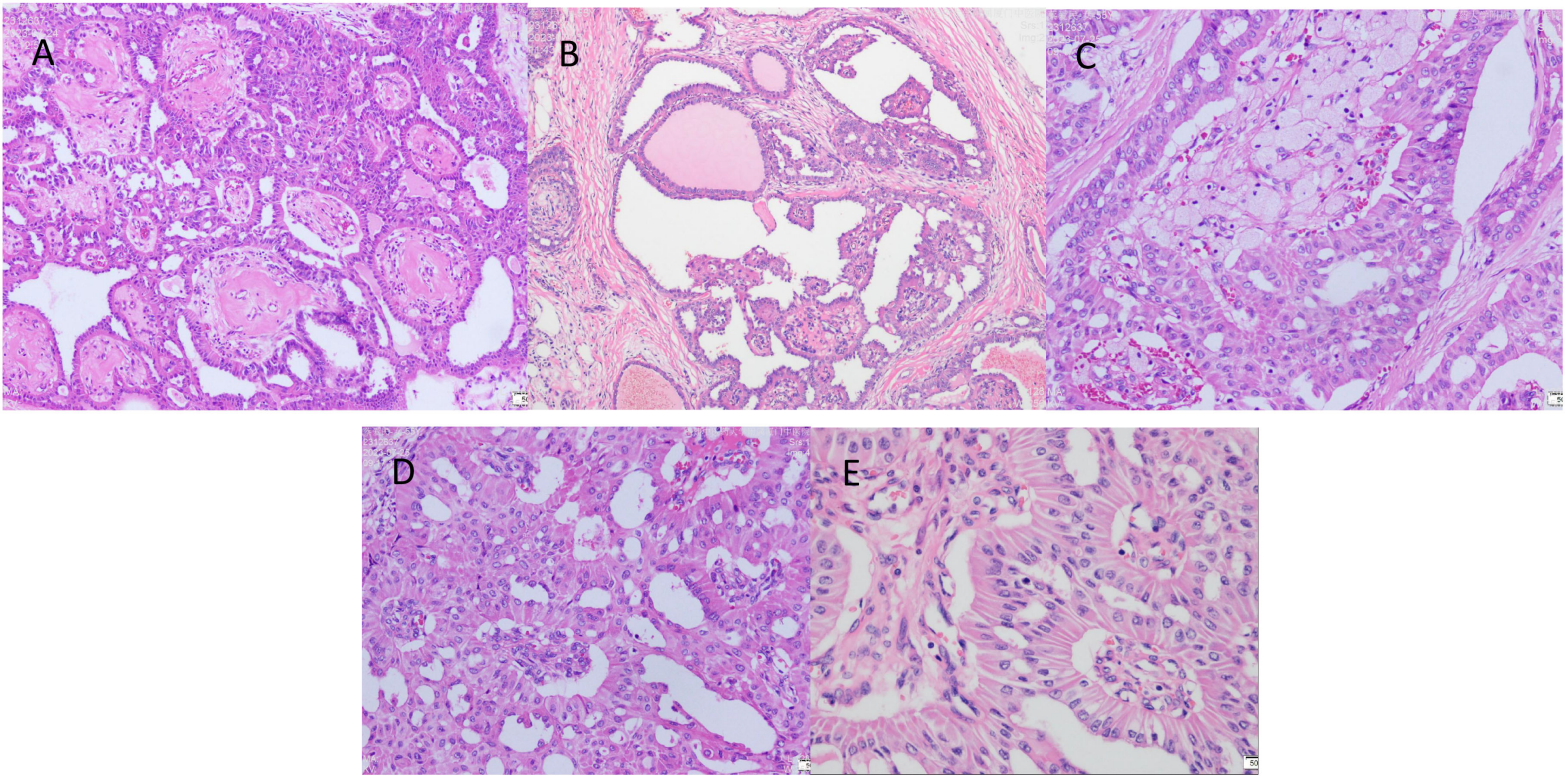
The postoperative pathological examination with haematoxylin and eosin (HE) staining. **(A)** Cancer cells arranged in a papillary structure (×100). **(B)** Cancer cells are arranged in a glandular structure, some of which are cystically dilated, similar to thyroid follicles, with individual glandular lumens containing gelatinous, homogeneous, strongly eosinophilic material, and interstitial fibrous connective tissue (×100). **(C)** Foam cell aggregates are seen in the interstitium of cancerous tissue (×200). **(D)** The surface of the papilla is covered with cancer cells in a cubic or high columnar shape, the nucleus is far from the base, the cytoplasm is reddish stained, and the interstitium is seen as a fibrovascular axis (×200). **(E)** Cancer cells are arranged in high columnar shape with mild nuclear morphology, eosinophilic granular cytoplasm, and nuclei are located away from the base at the luminal margin (×400).

The excised specimen was fixed in 10% formalin buffer and paraffin embedded. Sections were stained with haematoxylin, eosin and immunohistochemistry. Microscopically, the tumour tissue was seen to have a lobe-like structure, with papillary, sieve-like and glandular duct-like structures within the lobe, with cells arranged in a cubic and high columnar pattern, with nuclei distant from the base, slightly enlarged, ovoid, with thickened nuclear membranes and occasional nuclear grooves, with reddish-stained cytoplasm, interstitial fibroplasia, and foamy cells were seen in the foci (**Figures 3A–E**).

**Figure 4:**
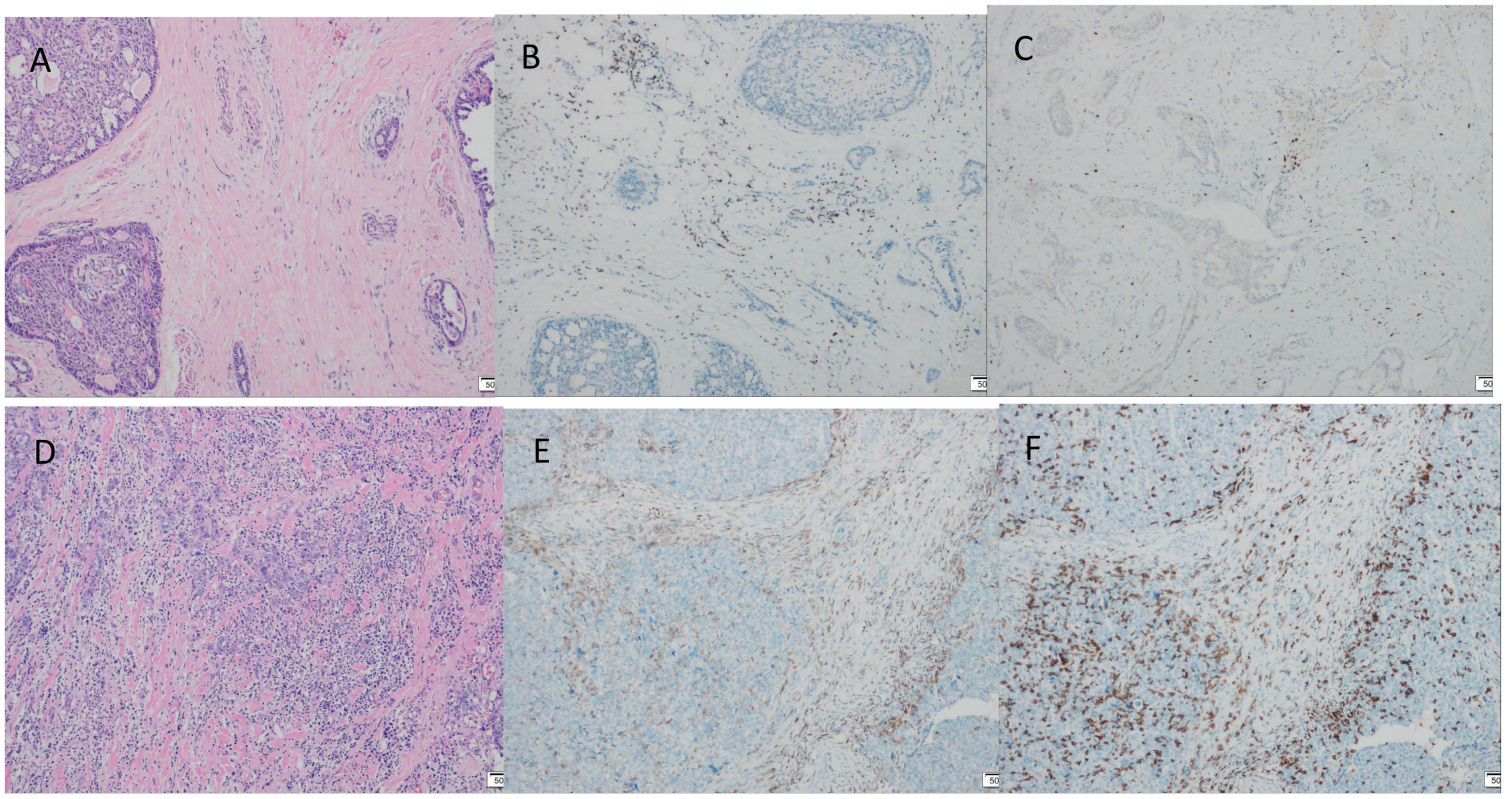
Tumour TILs infiltration assessment, TILs were stained by haematoxylin and eosin (HE) staining, CD4^+^ T lymphocytes, CD8^+^ T lymphocytes were stained by immunohistochemical (IHC) staining. **(A)** TILs < 10% (×100). **(B)** A small number of interstitial CD4^+^ T lymphocytes infiltrated (×100). **(C)** Small amount of interstitial CD8^+^ T lymphocyte infiltration (×100). **(D-F)** Common triple-negative breast cancer control. **(D)** TILs > 40% (×100). **(E)** CD4^+^ positive expression (×100). **(F)** CD8^+^ positive expression (×100).

**Figure 5:**
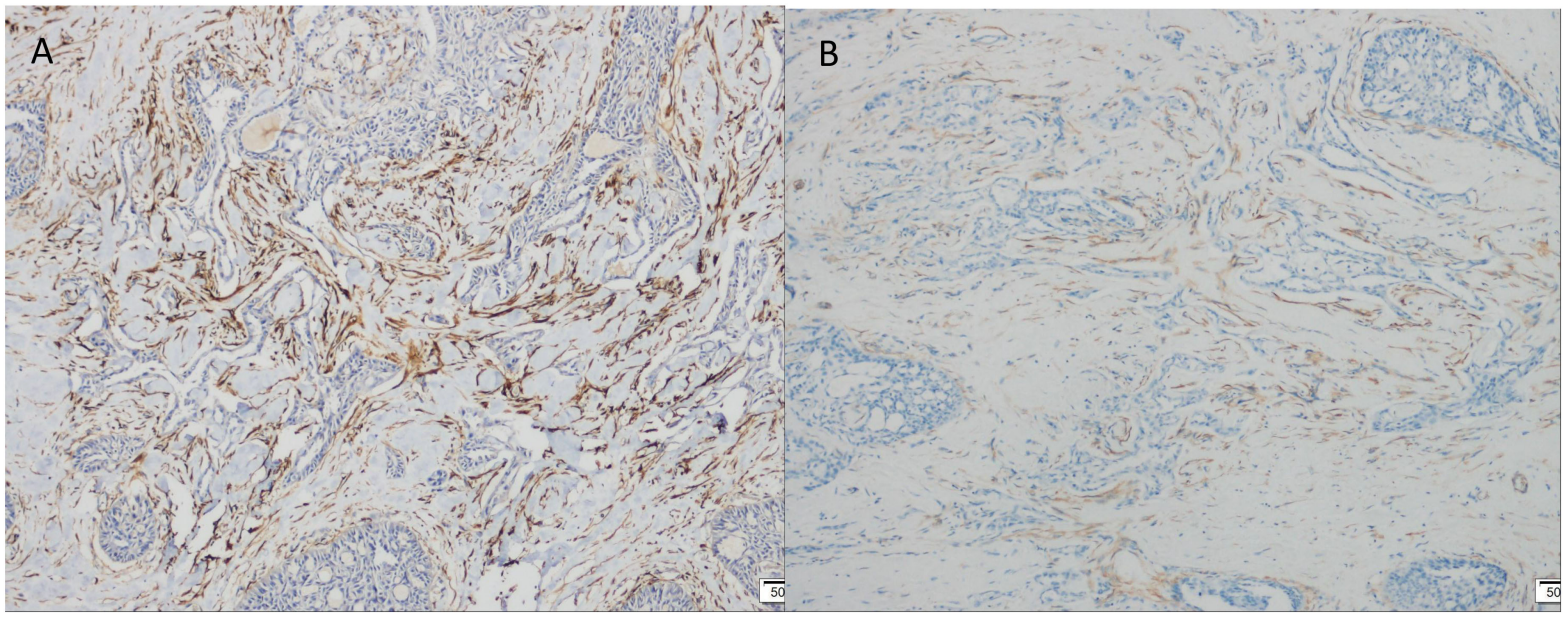
Immunohistochemical (IHC) staining. **(A)** FAP positive expression (×100). **(B)** SMA positive expression (×100).

**Figure 6:**
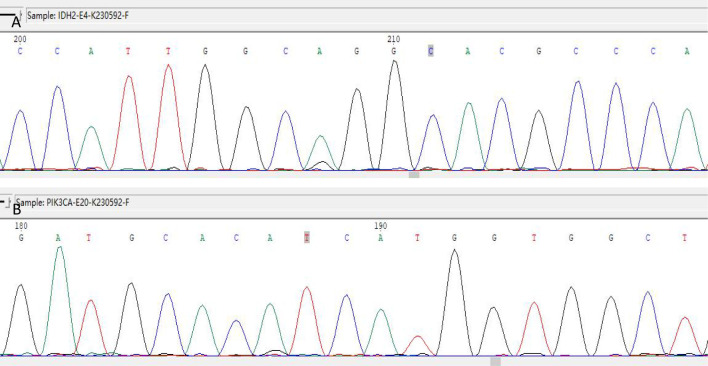
IDH2 R172 and PIK3CA H1047 mutations detected by Sanger sequencing. **(A)** IDH2 gene detected the c.514A > G (p.R172G) mutation. **(B)** The PIK3CA gene was detected with the c.3140A > G (p.H1047R) mutation.

Immunohistochemistry showed that TTF-1 and TG were negative, and GATA-3 was positive, indicating that the breast origin was not from the thyroid gland. P63 and Calponin were negative, indicating that the myoepithelium was absent and invasive, and the diagnosis of invasive ductal carcinoma was supported by the positivity of P120, E-cad, and CK8, and the negativity of ER, PR, and HER-2, which was TNBC. Calretinin positivity could support the diagnosis, CK5/6, CD56 positivity, EGFR, EMA, CD117, GCDFP-15, AR, Syn, CgA were all negative, KI67 was 10%, and the value-added index was low. For TILs and subpopulation detection, TILs were < 10% (**Figure 4A**), and the tumour interstitium was infiltrated by a small number of CD4^+^ and CD8^+^ T lymphocytes (**Figures 4B, C**). We compared it with common TNBC (**Figures 4D–F**). Detection of Cancer-Associated Fibroblasts (CAF) markers showed positivity for SMA and FAP (**Figures 5A, B**), and Sanger sequencing detected mutations in IDH2 (R172G) and PIK3CA (H1047R) genes (**Figures 6A, B**). Clinical staging was pT_2_N_0_M_0_, stage IIA, diagnosed according to the Chinese Society of Clinical Oncology (CSCO) Breast Cancer Guidelines 2023 (8).

The patient has received 4 cycles of post-operative chemotherapy and has been followed for 15 months and is currently disease-free.

### 2.2 Literature review

#### 2.2.1 Literature search results

**Figure 7:**
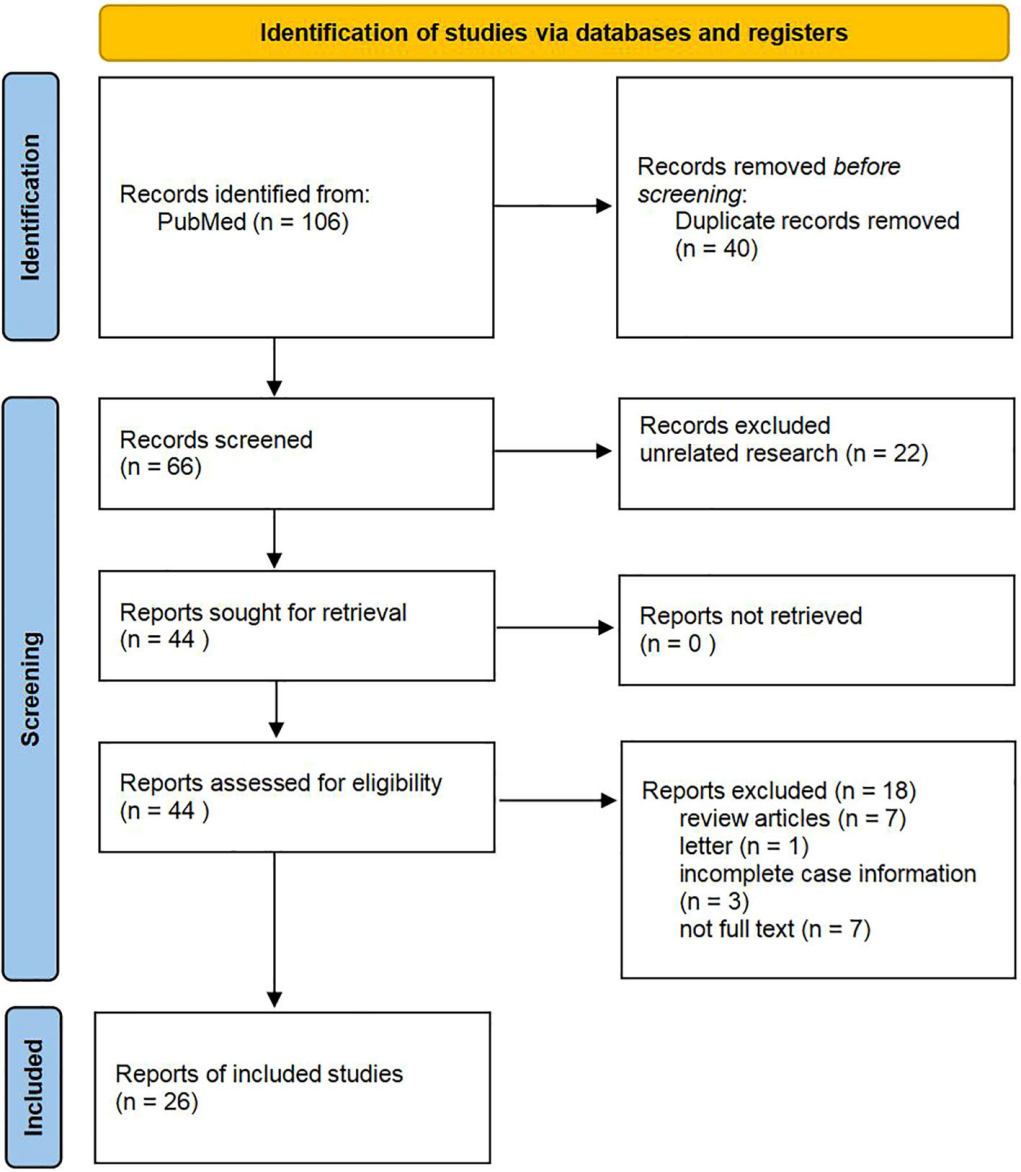
Flow diagram of study selection.

A bibliographic search of PubMed was performed using the following keywords: “Tall cell carcinoma with reversed polarity of the breast(TCCRP)” OR “Breast Tumor Resembling the Tall Cell Variant of Papillary Thyroid Carcinoma (BTPTC)” OR “Tall Cell Variant of Papillary Breast Carcinoma (TCVPBC)” OR “Solid Papillary Carcinoma with Reverse Polarity (SPCRP)” OR “Solid Papillary Breast Carcinomas Resembling the Tall Cell Variant of Papillary Thyroid Neoplasms (BPTC)”. A total of 106 papers were retrieved. We screened 66 remaining studies for eligible title and abstract after removing duplicates (n = 40), and 40 were excluded due to ineligibility or irrelevance (**Figure 7**). The abstract and title were read, and 22 articles were eliminated. After determining eligibility, 18 articles were excluded because 7 were “review articles”, 1 was “letter”, 3 were “incomplete case information”, and 7 were “not full text”. As a result, we finally included 26 studies. For each paper included, we recorded authors, demographics, clinical characteristics, treatment, and follow-up.

#### 2.2.2 Characteristics of included studies

A total of 26 relevant papers involving 96 patients were published, and we summarised the clinical data of the patients (**Table 1**). All reported patients were female, age range 40-85 years with a mean of 61.6 years, tumour size 0.6-5 cm with a mean of 1.9 cm, 61.5% of patients reported imaging data including Ultrasound, Mammogram and MRI, 44.8% of patients belonged to TNBC, and among the patients who underwent genetic testing so far, there were 59.4% had IDH2 mutations and 33.3% had PIK3CA mutations. Local excision was the mainstay of treatment, and metastasis was found in 4 cases during follow-up, 1 case had intramammary lymph node metastasis at the time of consultation, 1 case had axillary lymph node metastasis at the time of consultation, 1 case had axillary lymph node metastasis at the time of consultation and bone metastasis 32 months later, and 1 case had local recurrence and axillary lymph node metastasis 5 years after surgical excision. No fatal patients have been reported, and the follow-up time was 0-132 months, with a mean of 25.1 months.

**Table 1:** Summary of TCCRP case report clinical information.

Literature	Cases	Age (The average)	Tumour size (cm)	Tumour site (cases)	Lymph node metastasis	Imaging	TNBC	IDH2 mutation	PIK3CA mutation	Treatment	Follow-up (average month)
Eusebi et al.2003 (9)	5	56-74 (63)	0.8-2.0 (1.4)	L (2) R (2) NA (1)	UK	UK	0	ND	ND	Wide excision (3);quadrantectomy (1);UK (1)	26-108 (54),NED (4) UK (1)
Cameselle et al.2006 (10)	1	64	4.1	R	axillary lymph node	UK	0	ND	ND	Mastectomy+ALND+C/XRT/HT	32, bone metastases
Tosi et al.2007 (11)	4	45-80 (58)	2-5 (3)	R (4)	intramammary lymph node (1) No (3)	UK	0	ND	ND	Quadrantectomy (4)	3-10 (6.5)NED
Chang et al.2009 (12)	1	66	1.1	L	No	Ultrasound	0	ND	ND	quadrantectomy and SLN excision	12, NED
Masood et al.2012 (13)	1	57	3.7	L	No	Ultrasound,Mammogram	0	ND	ND	mastec- tomy	UK
Colella et al.2015 (14)	1	79	3	R	No	Ultrasound	1	ND	ND	mastectomy with ALND	18
Chiang et al.2016 (15)	13	51-79 (65)	0.6-1.8 (1.1)	L (8) R (5)	No	UK	6	10	8	S (1) S+XRT (2) S+C (1) S+XRT+C (1) NA (8)	12-77 (34) NED (7) UK (6)
Bhargava et al.2017 (16)	3	48-65 (63)	0.9-1.7 (1.3)	L (2) R (1)	UK	MRI (1)	UK	2	2	S (3)	0-19 (13) NED (2) UK (1)
Foschini et al.2017 (17)	13	48-85 (62.6)	0.6-2.5 (1.5)	L (7) R (6)	axillary lymph node (2) No (11)	Ultrasound,Mammogram	10	ND	ND	wide excision (11) wide excision+ALND (1) wide excision+XRT+C (1)	24-132 (71) Recurrence and lymph node metastasis (1) NED (10) UK (2)
Pitino et al.2017 (18)	1	65	0.7	R	No	Ultrasound,Mammogram	0	ND	ND	quadrantectomy +SLN excision	34,NED
Lozada et al.2018 (19)	6	58-85 (60)	0.6-2.1 (1.2)	UK	No	UK	6	6	4	UK	UK
Alsadoun et al.2018 (20)	9	52-75 (66)	0.9-4 (1.7)	L (5) R (4)	UK	UK	4	6	ND	quadrantectomy (8) quadrantectomy+SLN (1)	53 NED (1) UK (8)
Gai et al.2018 (21)	1	55	UK	R	No	Mammogram	1	ND	ND	simple mastectomy+SLNB	UK
Zhong et al.2019 (22)	9	63-79 (70)	0.7-1.8 (1.3)	R (5) L (4)	No	UK	4	7	6	UK	16-64 (32) NED (9)
Ding et al.2019 (23)	1	70	1.6	L	No	Mammogram	1	1	1	quadrantectomy	9,NED
Haefliger et al.2020 (24)	1	60	0.8	R	No	Mammogram	1	1	1	quadrantectomy+SLN excision	8,NED
Pareja et al.2020 (25)	14	46-85 (63)	0.6-2.6 (1.3)	UK	No	UK	UK	14	7	quadrantectomy (2) quadrantectomy+XRT (3) mastectomy (1) UK (8)	UK
Jassim et al.2021 (26)	1	40	5.5	R	No	Mammogram	0	1	ND	mastectomy+ALND	6,NED
Zhang et al.2021 (27)	1	45	1	L	No	Ultrasound, Mammogram	1	1	1	wide local excision+SLNB	UK
Trihia et al.2021 (28)	1	71	0.9	R	No	Mammogram	1	ND	ND	Quadrantectomy+SLNB	UK
Wei et al.2021 (29)	2	70-72 (71)	1.6-2.2 (1.9)	R (2)	No	Ultrasound	2	2	2	mastectomy (1) mastectomy+SLNB (1)	6-33 (19.5),NED
Cui et al.2021 (30)	1	62	2	L	No	Ultrasound	1	1	ND	mastectomy	6,NED
Sasaki et al.2022 (31)	3	44-71 (57)	0.4-1.4 (0.9)	L (2) R (1)	No	UK	1	3	ND	Wide excision+SLNB (3)	8-19 (14),NED
Lee et al.2023 (32)	1	64	1.6	R	No	Ultrasound,Mammogram,MRI	1	ND	ND	Quadrantectomy+SLNB	12,NED
Elghobashy et al.2023 (33)	1	41	2.2	L	No	Ultrasound,Mammogram	1	1	ND	Quadrantectomy+SLNBX+XRT	19,NED
Lei et al.2024 (34)	1	65	2	L	No	Ultrasound,Mammogram,MRI	1	1	ND	Quadrantectomy	48,NED

ALND, axillary lymph node dissection; C, chemotherapy; HT, endocrine therapy; L, left; ND, not done; NED, disease-free survival; R, right; SLN, sentinel lymph node; SLNB, sentinel lymph node biopsy; UK, unknown; XRT, radiation therapy.

## 3 Discussion

TCCRP is a rare subtype of breast cancer which was first identified by Eusebi in 2003 (9) and was initially named Breast Tumour Resembling the Tall Cell Variant of Papillary Thyroid Cancer (BTPTC) because of the similarity of the pathologic features to the high cell variant of Papillary Thyroid Carcinoma (BTPTC), an important morphological feature of this tumour is the proximity of the cell nuclei to the luminal surface of the gland, known as polarity flip. In 2019, the WHO Blue Book classification of breast tumours (5th edition) included this rare tumour (35) and named Tall Cell Carcinoma with Reversed Polarity (TCCRP), which was recognised as an independent subgroup included in rare and salivary gland-type tumours.

In this paper, we study and summarise the relevant TCCRP literature in recent years: patients are mainly middle-aged and elderly women, usually consulting for a palpable breast lump or screening mammogram, Ultrasound and Mammogram are predominantly used in the imaging data, but the description of BI-RADS grading is missing in many cases, and TNBC has the highest percentage in TCCRP, and a few patients have weak positivity of the hormone receptors were weakly positive, and the remaining patients lacked HER-2 testing for definitive molecular staging. In immunohistochemistry, breast origin markers GCDFP-15 and GATA-3 were frequently strongly positively expressed, thyroid papillary carcinoma markers TTF-1 and TG were negative, the majority of TCCRP had a Ki67 proliferative index of less than 10% indicating a better prognosis, CK5/6 was usually positive, and p63 was usually negative suggesting a certain degree of aggressiveness. In terms of diagnosis, early studies have shown that TCCRP does not have molecular genetic alterations typical in papillary thyroid carcinoma, such as BRAF and RET gene mutations, Chiang et al. (15) found that mutations in the IDH2 and PIK3CA genes are characteristic mutations in TCCRP, and Alsadoun et al. (20) suggested that Calretinin is also a useful TCCRP marker, as Calretinin expression is rare in low-grade breast cancers, whereas high-grade breast cancers with poorer prognosis are usually positive for Calretinin. It is now generally accepted that TCCRP has an inert biological behaviour and a good prognosis, and in terms of treatment, some scholars support the absence of active clinical management, while others have proposed to consider the use of IDH2 inhibitors.

The clinical, pathologic, and immunohistochemical features of our TCCRP case were consistent with previous reports of a 54-year-old woman with a painless breast mass that presented clinically and had been present for 10 years, similar to the history reported by Tosi (11). However, the Mammogram in this patient suggested benign, and it was considered that the diagnosis was missed due to the extremely dense type of the patient’s gland and the low sensitivity of the Mammogram for the mass, and Foschini (17) also suggested that the lesions were mostly interpreted as benign on Mammogram examination or Ultrasound because of the regularity of their margins. The diagnosis of TCCRP on the basis of puncture pathology is difficult as it shows columnar to cuboidal tumour cells, with nuclei seen in very few areas away from the base. Postoperative pathology had typical features of TCCRP, with papillary, glandular-like structure of cancer cells, some of which were cystically dilated, similar to thyroid follicles, and the surface of the papillae was covered with cancer cells in a cuboidal or high columnar shape, with nuclei far away from the base. The interstitium of the cancerous tissue was slender fibrous connective tissue with fibrovascular axons, and foam cell aggregates were seen. Immunohistochemistry was triple-negative, KI67 was 10% with a good prognosis, Calretinin was positive, and genetic testing suggested IDH2 and PIK3CA mutations. The current follow-up period is 15 months and the patient will be followed for a long time in the future. In addition, there are no studies on TILs infiltration in the TME of TCCRP, and we compared the present case with a case of common TNBC according to the international guidelines on TILs detection published by the International Immuno-Oncology Biomarker Working Group in 2014 (36), with immunohistochemical detection of CD4^+^ T cell and CD8^+^ T cell expression to first explore the characteristics of TCCRP TILs infiltration.

Breast cancer used to be regarded as a low-immunogenic tumour, but in recent years, studies have revealed the presence of TILs infiltration in the stroma of TNBC, which is attributed to the high degree of genetic instability and mutational load leading to the production of a large number of neoantigens (37), which are capable of strong immune responses and strong immunoediting effects. And the higher the degree of TILs infiltration the better the prognosis, clinical studies have shown that high levels of specific phenotypic TILs (CD4^+^, CD8^+^) can positively predict the long-term prognosis of TNBC (38). It has been suggested that immune-rich TNBC may be under immune surveillance, which consistently eliminates many immunogenic clones and thus reduces clonal heterogeneity and achieves a balanced state of immune surveillance and clonal heterogeneity, while TNBC with low TILs have a poor prognosis and may represent the onset of immune escape and tumour evolution toward greater clonal heterogeneity and genomic diversity (39). Our case of TCCRP was a low-immunogenic tumour with low levels of TILs but good prognosis, a specific TNBC subtype. The reason may be that TCCRP has a more stable genome with low somatic copy number as well as clonal heterogeneity, and there was no significant neoantigen production, which resulted in the absence of a strong immune response. In addition, we observed a large amount of fibrous connective tissue in the TCCRP mesenchyme, and the fibrotic mesenchyme was able to inhibit anti-tumour immune responses and allow the tumour to undergo immune escape. Fibroblasts are the main cells of the tumour mesenchyme that can be induced by cancer cells to become Cancer-Associated Fibroblasts (CAF), and these fibroblasts usually express cytoskeletal proteins (α-SMA) or fibroblast activation proteins (FAP), and it is now proposed that CAFs can establish an immune-suppressing by interacting with the TME components and thus establishing an immune-suppressive microenvironment (40). CAF-enriched tumours exhibit an immunocold tumour microenvironment, and transcriptomics, flow cytometry, and quantitative histopathology analyses have demonstrated a negative correlation between CAF density and the anti-tumour phenotype of CD8^+^ T cells (41), which is consistent with the characteristics of the TCCRP in this case. In addition, CAF was able to promote the occurrence of M2 polarisation of tumour-associated macrophages, as well as a significant increase in the proportion of other immunosuppressive cells (regulatory T cells (Treg) and myeloid-derived suppressor cells (MDSC)) in TME, resulting in an immunosuppressive microenvironment (42). Four main subpopulations of CAF have been identified, which are categorised as CAF-S1 to CAF-S4 based on the expression of differentially activated markers, TNBC are generally enriched in CAF-S1 or CAF-S4, and FAP is expressed only in the CAF-S1 subpopulation (43). In this case, FAP was positive for SMA, which belongs to the CAF-S1 subpopulation, and CAF-S1 has an immunosuppressive function, and targeting of CAF may also help to allow more immune infiltration and restore immunosuppression. It has been shown that tumours in CAF receptor Endo180 knockout mice exhibit increased CD8^+^ T cell infiltration and enhanced sensitivity to immune checkpoint blockade (ICB) (41). FAP can also be used as a therapeutic target, for example FAPI-04 can be used to target breast cancers with high levels of activated fibroblasts (44). We hypothesised that targeting fibroblast subpopulations to improve clinical response to immunotherapy may be an effective treatment modality for TCCRP. It is also important to note that our findings are preliminary, as the 15-month follow-up is shorter than the mean of 25.1 months, and longer follow-up is needed to validate our findings. We have limitations in studying the immune characteristics of TME in our TCCRP case, CD4^+^ T cells and CD8^+^ T cells in TILs contain multiple cell subpopulations, which need to be differentiated on the basis of surface markers and secreted cytokines, and each subpopulation plays a different role in the immune response, for example, Th1 cells promote tumour killing, Treg suppress the immune response, Th1 cell markers include CD28, CD44, secrete IFN-γ and the Treg cell marker is FOXP3. In addition, the expression of other immune cells in the TME has an association with breast cancer prognosis. For example, there was a significant independent positive correlation between higher mature Tumour-Infiltrating Dendritic Cells (DCs) and longer recurrence-free survival (RFS) (45). Infiltration of tumour-associated macrophages (TAM) is usually associated with poor clinicopathological features, and a retrospective study had reported a poor prognostic role for M2-polarised TAM in breast cancer patients (46). There was also a study that preliminarily showed that increased natural killer (NK) cell expression was associated with improved RFS (47). Overall, in addition to TILs expression, the infiltration of other immune cells in the TME is also an important study to assess the prognosis of the tumour, and we initially only examined the infiltration of TILs, which has some limitations and does not represent the overall immune infiltration of the TCCRP. Subsequently, flow cytometry and single cell sequencing techniques can be used to further investigate the immune profile of TME in TCCRP, as well as the crosstalk between CAF subpopulations and immune cells to better understand the immune profile of TCCRP.

## 4 Conclusion

TCCRP is a rare type of TNBC with inert biological behaviour and good prognosis, the typical pathological feature is the papillary morphology of the nuclei away from the base. This case has the feature of low TILs infiltration, which may be a characteristic of TCCRP, the presence of CAF in the interstitium of the tumour in this case may have suppressed anti-tumour immunity to some extent, and further studies are needed regarding the immune characteristics of the TME in TCCRP.

## Data Availability

The original contributions presented in the study are included in the article/supplementary material. Further inquiries can be directed to the corresponding author.

## References

[1] Bray F Laversanne M Sung H Ferlay J Siegel RL Soerjomataram I . Global cancer statistics 2022: GLOBOCAN estimates of incidence and mortality worldwide for 36 cancers in 185 countries. CA Cancer J Clin. (2024) 74:229–63. doi: 10.3322/caac.21834 38572751

[2] Morris GJ Naidu S Topham AK Guiles F Xu Y McCue P . Differences in breast carcinoma characteristics in newly diagnosed African-American and Caucasian patients: a single-institution compilation compared with the National Cancer Institute's Surveillance, Epidemiology, and End Results database. Cancer. (2007) 110:876–84. doi: 10.1002/cncr.22836 17620276

[3] Denkert C Liedtke C Tutt A von Minckwitz G . Molecular alterations in triple-negative breast cancer-the road to new treatment strategies. Lancet. (2017) 389:2430–42. doi: 10.1016/S0140-6736(16)32454-0 27939063

[4] Debien V De Caluwé A Wang X Piccart-Gebhart M Tuohy VK Romano E . Immunotherapy in breast cancer: an overview of current strategies and perspectives. NPJ Breast Cancer. (2023) 9:7. doi: 10.1038/s41523-023-00508-3 36781869 PMC9925769

[5] Leon-Ferre RA Jonas SF Salgado R Loi S de Jong V Carter JM . Tumor-infiltrating lymphocytes in triple-negative breast cancer. JAMA. (2024) 331:1135–44. doi: 10.1001/jama.2024.3056 PMC10988354 38563834

[6] Stanton SE Disis ML . Clinical significance of tumor-infiltrating lymphocytes in breast cancer. J Immunother Cancer. (2016) 4:59. doi: 10.1186/s40425-016-0165-6 27777769 PMC5067916

[7] Denkert C von Minckwitz G Darb-Esfahani S Lederer B Heppner BI Weber KE . Tumour-infiltrating lymphocytes and prognosis in different subtypes of breast cancer: a pooled analysis of 3771 patients treated with neoadjuvant therapy. Lancet Oncol. (2018) 19:40–50. doi: 10.1016/S1470-2045(17)30904-X 29233559

[8] Guidelines Working Committee of the Chinese Society of Clinical Oncology 2023 . Chinese Society of Clinical Oncology (CSCO) Breast Cancer Guidelines 2023. Beijing: People's Medical Publishing House (2023).

[9] Eusebi V Damiani S Ellis IO Azzopardi JG Rosai J . Breast tumor resembling the tall cell variant of papillary thyroid carcinoma: report of 5 cases. Am J Surg Pathol. (2003) 27:1114–8. doi: 10.1097/00000478-200308000-00008 12883243

[10] Cameselle-Teijeiro J Abdulkader I Barreiro-Morandeira F Ruiz-Ponte C Reyes-Santías R Chavez E . Breast tumor resembling the tall cell variant of papillary thyroid carcinoma: a case report. Int J Surg Pathol. (2006) 14:79–84. doi: 10.1177/106689690601400116 16501842

[11] Tosi AL Ragazzi M Asioli S Del Vecchio M Cavalieri M Eusebi LH . Breast tumor resembling the tall cell variant of papillary thyroid carcinoma: report of 4 cases with evidence of Malignant potential. Int J Surg Pathol. (2007) 15:14–9. doi: 10.1177/1066896906295689 17172492

[12] Chang SY Fleiszer DM Mesurolle B El Khoury M Omeroglu A . Breast tumor resembling the tall cell variant of papillary thyroid carcinoma. Breast J. (2009) 15:531–5. doi: 10.1111/j.1524-4741.2009.00773.x 19594763

[13] Masood S Davis C Kubik MJ . Changing the term "breast tumor resembling the tall cell variant of papillary thyroid carcinoma" to "tall cell variant of papillary breast carcinoma. Adv Anat Pathol. (2012) 19:108–10. doi: 10.1097/PAP.0b013e318249d090 22313838

[14] Colella R Guerriero A Giansanti M Sidoni A Bellezza G . An additional case of breast tumor resembling the tall cell variant of papillary thyroid carcinoma. Int J Surg Pathol. (2015) 23:217–20. doi: 10.1177/1066896914536222 24868004

[15] Chiang S Weigelt B Wen HC Pareja F Raghavendra A Martelotto LG . IDH2 mutations define a unique subtype of breast cancer with altered nuclear polarity. Cancer Res. (2016) 76:7118–29. doi: 10.1158/0008-5472.CAN-16-0298 PMC5502804 27913435

[16] Bhargava R Florea AV Pelmus M Jones MW Bonaventura M Wald A . Breast tumor resembling tall cell variant of papillary thyroid carcinoma: A solid papillary neoplasm with characteristic immunohistochemical profile and few recurrent mutations. Am J Clin Pathol. (2017) 147:399–410. doi: 10.1093/ajcp/aqx016 28375433

[17] Foschini MP Asioli S Foreid S Cserni G Ellis IO Eusebi V . Solid papillary breast carcinomas resembling the tall cell variant of papillary thyroid neoplasms: A unique invasive tumor with indolent behavior. Am J Surg Pathol. (2017) 41:887–95. doi: 10.1097/PAS.0000000000000853 28418993

[18] Pitino A Squillaci S Spairani C Rassu PC Cosimi MF . Tall cell variant of papillary breast carcinoma: an additional case with review of the literature. Pathologica. (2017) 109:162–7. 29154377

[19] Lozada JR Basili T Pareja F Alemar B Paula ADC Gularte-Merida R . Solid papillary breast carcinomas resembling the tall cell variant of papillary thyroid neoplasms (solid papillary carcinomas with reverse polarity) harbour recurrent mutations affecting IDH2 and PIK3CA: a validation cohort. Histopathology. (2018) 73:339–44. doi: 10.1111/his.13522 PMC6783257 29603332

[20] Alsadoun N MacGrogan G Truntzer C Lacroix-Triki M Bedgedjian I Koeb MH . Solid papillary carcinoma with reverse polarity of the breast harbors specific morphologic, immunohistochemical and molecular profile in comparison with other benign or Malignant papillary lesions of the breast: a comparative study of 9 additional cases. Mod Pathol. (2018) 31:1367–80. doi: 10.1038/s41379-018-0047-1 29785016

[21] Gai L Done SJ Cook D Denic N Erivwo P Voisey K . Breast tumour resembling tall cell variant of papillary thyroid carcinoma: case presentation (in a patient with Lynch syndrome). J Clin Pathol. (2018) 71:1031–2. doi: 10.1136/jclinpath-2018-205337 29982234

[22] Zhong E Scognamiglio T D'Alfonso T Song W Tran H Baek I . Breast tumor resembling the tall cell variant of papillary thyroid carcinoma: molecular characterization by next-generation sequencing and histopathological comparison with tall cell papillary carcinoma of thyroid. Int J Surg Pathol. (2019) 27:134–41. doi: 10.1177/1066896918800779 30227763

[23] Ding LM Hu HX Wang YJ Ji D Ni LY Sun ZH . Tall cell variant of papillary breast carcinoma: report of a case. Zhonghua Bing Li Xue Za Zhi. (2019) 48:815–7. doi: 10.3760/cma.j.issn.0529-5807 31594051

[24] Haefliger S Muenst S Went P Bihl M Dellas S Weber WP . Tall cell carcinoma of the breast with reversed polarity (TCCRP) with mutations in the IDH2 and PIK3CA genes: a case report. Mol Biol Rep. (2020) 47:4917–21. doi: 10.1007/s11033-020-05553-w 32474846

[25] Pareja F da Silva EM Frosina D Geyer FC Lozada JR Basili T . Immunohistochemical analysis of IDH2 R172 hotspot mutations in breast papillary neoplasms: applications in the diagnosis of tall cell carcinoma with reverse polarity. Mod Pathol. (2020) 33:1056–64. doi: 10.1038/s41379-019-0442-2 PMC7286791 31896809

[26] Jassim M Premalata CS Okaly G Srinivas C . Tall cell carcinoma with reverse polarity of breast: report of a case with unique morphologic and molecular features. Turk Patoloji Derg. (2021) 37:183–8. doi: 10.5146/tjpath.2020.01511 PMC10512679 33021737

[27] Zhang X Wu H Wang Z Zhou Y Mao F Lin Y . Tall cell carcinoma of the breast with reverse polarity: case report with gene sequencing and literature review. Gland Surg. (2021) 10:837–43. doi: 10.21037/gs-20-695 PMC7944084 33708566

[28] Trihia HJ Lampropoulos P Karelis L Souka E Galanopoulos G Provatas I . Tall cell carcinoma with reversed polarity: A case report of a very rare breast tumor entity and mini-review. Breast J. (2021) 27:369–76. doi: 10.1111/tbj.14165 33527653

[29] Wei Y Ding L Song X Tian X Min N Guan Q . Tall cell carcinoma with reversed polarity: case report with gene sequencing and literature review. Gland Surg. (2021) 10:3147–54. doi: 10.21037/gs-21-591 PMC8637072 34926230

[30] Cui LJ Zhao YF He J Lai BA He ZZ . Tall cell carcinoma with reverse polarity of breast with papillary thyroid carcinoma: report of a case. Zhonghua Bing Li Xue Za Zhi. (2021) 50:1299–301. doi: 10.3760/cma.j.cn112151-20210322-00222 34719179

[31] Sasaki E Iwakoshi A Satake T Nakajima K Kobayashi M Ando Y . The diagnostic utility of IDH2 R172 immunohistochemistry in tall cell carcinoma with reversed polarity of the breast. Appl Immunohistochem Mol Morphol. (2022) 30:654–61. doi: 10.1097/PAI.0000000000001074 36222504

[32] Lee NY Chang YW Lee EJ Jin YM . Tall cell carcinoma with reversed polarity of breast: Sonographic and magnetic resonance imaging findings. J Clin Ultrasound. (2023) 51:494–7. doi: 10.1002/jcu.23280 35904337

[33] Elghobashy M Jenkins S Shulman Z O'Neil A Kouneli S Shaaban AM . Tall cell carcinoma with reversed polarity: case report of a rare special type of breast cancer and review of the literature. Biomedicines. (2023) 11:2376. doi: 10.3390/biomedicines11092376 37760817 PMC10525258

[34] Lei Z Wang YX Wang ZY Yang CG Pan GQ . Case report: Tall cell carcinoma with reversed polarity of the breast: an additional case and review of the literature. Front Oncol. (2024) 14:1302196. doi: 10.3389/fonc.2024.1302196 38434689 PMC10904622

[35] Tumours Editorial Board of the WHO Classification . Classification of Breast Tumours. 5th ed. Lyon: IARC Press (2019).

[36] Salgado R Denkert C Demaria S Sirtaine N Klauschen F Pruneri G . The evaluation of tumor-infiltrating lymphocytes (TILs) in breast cancer: recommendations by an International TILs Working Group 2014. Ann Oncol. (2015) 26:259–71. doi: 10.1093/annonc/mdu450 PMC6267863 25214542

[37] Dieci MV Griguolo G Miglietta F Guarneri V . The immune system and hormone-receptor positive breast cancer: Is it really a dead end? Cancer Treat Rev. (2016) 46:9–19. doi: 10.1016/j.ctrv.2016.03.011 27055087

[38] Gao G Wang Z Qu X Zhang Z . Prognostic value of tumor-infiltrating lymphocytes in patients with triple-negative breast cancer: a systematic review and meta-analysis. BMC Cancer. (2020) 20:179. doi: 10.1186/s12885-020-6668-z 32131780 PMC7057662

[39] Karn T Jiang T Hatzis C Sänger N El-Balat A Rody A . Association between genomic metrics and immune infiltration in triple-negative breast cancer. JAMA Oncol. (2017) 3:1707–11. doi: 10.1001/jamaoncol PMC5824276 28750120

[40] Kalluri R . The biology and function of fibroblasts in cancer. Nat Rev Cancer. (2016) 16:582–98. doi: 10.1038/nrc.2016.73 27550820

[41] Jenkins L Jungwirth U Avgustinova A Iravani M Mills A Haider S . Cancer-associated fibroblasts suppress CD8+ T-cell infiltration and confer resistance to immune-checkpoint blockade. Cancer Res. (2022) 82:2904–17. doi: 10.1158/0008-5472.CAN-21-4141 PMC9379365 35749591

[42] Takahashi H Sakakura K Kudo T Toyoda M Kaira K Oyama T . Cancer-associated fibroblasts promote an immunosuppressive microenvironment through the induction and accumulation of protumoral macrophages. Oncotarget. (2017) 8:8633–47. doi: 10.18632/oncotarget.14374 PMC5352428 28052009

[43] Costa A Kieffer Y Scholer-Dahirel A Pelon F Bourachot B Cardon M . Fibroblast heterogeneity and immunosuppressive environment in human breast cancer. Cancer Cell. (2018) 33:463–479.e10. doi: 10.1016/j.ccell.2018.01.011 29455927

[44] Lindner T Loktev A Altmann A Giesel F Kratochwil C Debus J . Development of quinoline-based theranostic ligands for the targeting of fibroblast activation protein. J Nucl Med. (2018) 59:1415–22. doi: 10.2967/jnumed.118.210443 29626119

[45] Iwamoto M Shinohara H Miyamoto A Okuzawa M Mabuchi H Nohara T . Prognostic value of tumor-infiltrating dendritic cells expressing CD83 in human breast carcinomas. Int J Cancer. (2003) 104:92–7. doi: 10.1002/ijc.10915 12532424

[46] Tiainen S Tumelius R Rilla K Hamalainen K Tammi M Tammi R . High numbers of macrophages, especially M2-like (CD163-positive), correlate with hyaluronan accumulation and poor outcome in breast cancer. Histopathology. (2015) 66:873–83. doi: 10.1111/his.12607 25387851

[47] Ascierto ML Idowu MO Zhao Y Khalak H Payne KK Wang XY . Molecular signatures mostly associated with NK cells are predictive of relapse free survival in breast cancer patients. J Transl Med. (2013) 11:145. doi: 10.1186/1479-5876-11-145 23758773 PMC3694475

